# Localized amyloidosis of the epididymis: a previously unreported phenomenon

**DOI:** 10.1186/s13000-017-0646-z

**Published:** 2017-08-04

**Authors:** Lucio Díaz-Flores, Ricardo Gutiérrez, Ma. del Pino García, Manuel Jose Gayoso, Jose Luis Carrasco, Lucio Díaz-Flores, Hugo Álvarez-Argüelles

**Affiliations:** 1 Department of Basic Medical Sciences, Faculty of Medicine, University of La Laguna, Ofra-La Cuesta, s/n, La Laguna, 38071 Tenerife, Islas Canarias Spain; 2 Department of Pathology, Hospiten® Hospitals, Tenerife, Spain; 3 Department of Cell Biology and Pharmacology, Faculty of Medicine, University of Valladolid, Valladolid, Spain

**Keywords:** Epididymis, Amyloid, Amyloidosis

## Abstract

**Background:**

Localized amyloidosis has not been documented in the epididymis; we report this phenomenon for the first time.

**Case presentation:**

The first aim of this work is to report three cases of localized epididymal amyloidosis. Two cases were clinically detected as epididymal nodules, and a third after reviewing 120 epididymides obtained with neighbouring pathological processes. Amyloid deposits showed Congo red positivity, with yellow-green birefringence, and immunohistochemical expression for light chains kappa and lambda, transthyretin, amyloid P and cytokeratin AE1 AE3. No immunoreactivity for amyloid A was seen. Amyloid deposit location was intraluminal, with partial or total loss of the epididymal epithelium and subsequent passage to the interstitium, forming large masses. No amyloid deposits were observed around blood vessels. A secondary objective was to explore in normal epididymis the amyloid tested in epididymal amyloidosis. In normal epididymides, expression of amyloid P and transthyretin was detected in the apical surface of epithelial cells. Amyloid P also showed strong expression in spermatozoa.

**Conclusion:**

We contribute the existence of localized epididymal amyloidosis, which presents a distinctive, initial intratubular location, where there is a unique proteome and where functional amyloids act during sperm maturation.

## Background

In anatomical regions of the male reproductive system that contribute to the transport, maturation and/or required fluid medium of spermatozoa, localized amyloidosis has been pointed out in seminal vesicles, vasa deferentia and ejaculatory ducts [1–9]. The objective of this work is to report localized amyloidosis in the epididymis for the first time. In addition, we study the following: a) amyloid deposit distribution in the epididymis, to assess where the deposits are formed and b) the presence in normal epididymides of the amyloids tested in our cases of epididymal amyloidosis. Our observations indicate that epididymal amyloidosis is organ-limited, with a distinctive initial location (intratubular).

## Case presentation

After observing two cases (Cases 1 and 2) of pseudotumoral epididymal amyloidosis, epididymides (n: 120) were examined for the presence of pathologic amyloid deposits and for amyloid detection. A new case (Case 3) of subclinical amyloidosis was obtained in this review. All patients were Caucasian, and the relevant findings of the cases are shown in Table 1. Evidence of systemic amyloidosis, paraproteinemia, or underlying plasma cell dyscrasia was not demonstrated. Finally, the amyloids tested in epididymal amyloidosis were also checked in seven normal epididymides. The study was carried out in accordance with the code of ethics of the World Medical Association.

**Table 1:** Characteristics of reported cases and antibodies used for immunohistochemistry

Case	Age (years)	Presentation and resulting diagnosis	Larger diameter (cm)	Contralateral epididymal exploration	Operation	Follow-up (months)	IHC Primary antibodies used
1	77	Nodule in the left epididymis Result: Epididymal amyloidosis	1.4	Thickened	Nodule removal	48 (Free of disease)	Light chain λ Dako [D: 1:50] Light chain κ Dako [D: 1:50] Transthyretin Dako [D: 1:600] Amyloid P Abcam [D: 1:50] Amyloid A Dako [D: 1:50] CK AE1 AE3 Dako [D: 1:100] EMA Dako [D: 1:100] CD68 Dako [D: 1:100] CD34 Dako [D: 1:50] αSMA Dako [D: 1:50]
2	72	Nodule in the right epididymis Result: Epididymal amyloidosis	1.6	NED	Nodule removal	9 (Free of disease)	
3	67	Left scrotal swelling for 4 years. Physical examination: a firm, non-reducible mass. Result: Paratesticular liposarcoma and Epididymal amyloidosis without tumour involvement	0.7 (size refers only to epididymal amyloidosis)	NA	Radical Orchiectomy	NA	

*IHC* Immunohistochemistry, *NED* No evidence of disease, *NA* Not available, *CK* Cytokeratin, *EMA* Epithelial membrane antigen

Histologic sections were stained with Haemotoxylin and Eosin (H&E), Congo red, Periodic Acid-Schiff (PAS) and Wilder’s reticulin stain. After Congo red staining, the sections were also viewed under polarized light. Immunohistochemical study included the primary antibodies listed in Table 1.

## Results

### General characteristics of epididymal amyloidosis

In cases 1 and 2 of epididymal amyloidosis, the surgically removed nodules were firm, yellowish grey in colour, and 1.4 and 1.6 cm in size, respectively. Case 3 (obtained after the microscopic review of 120 epididymides) showed a larger diameter of 0.7 cm (Table 1).

**Fig. 1:**
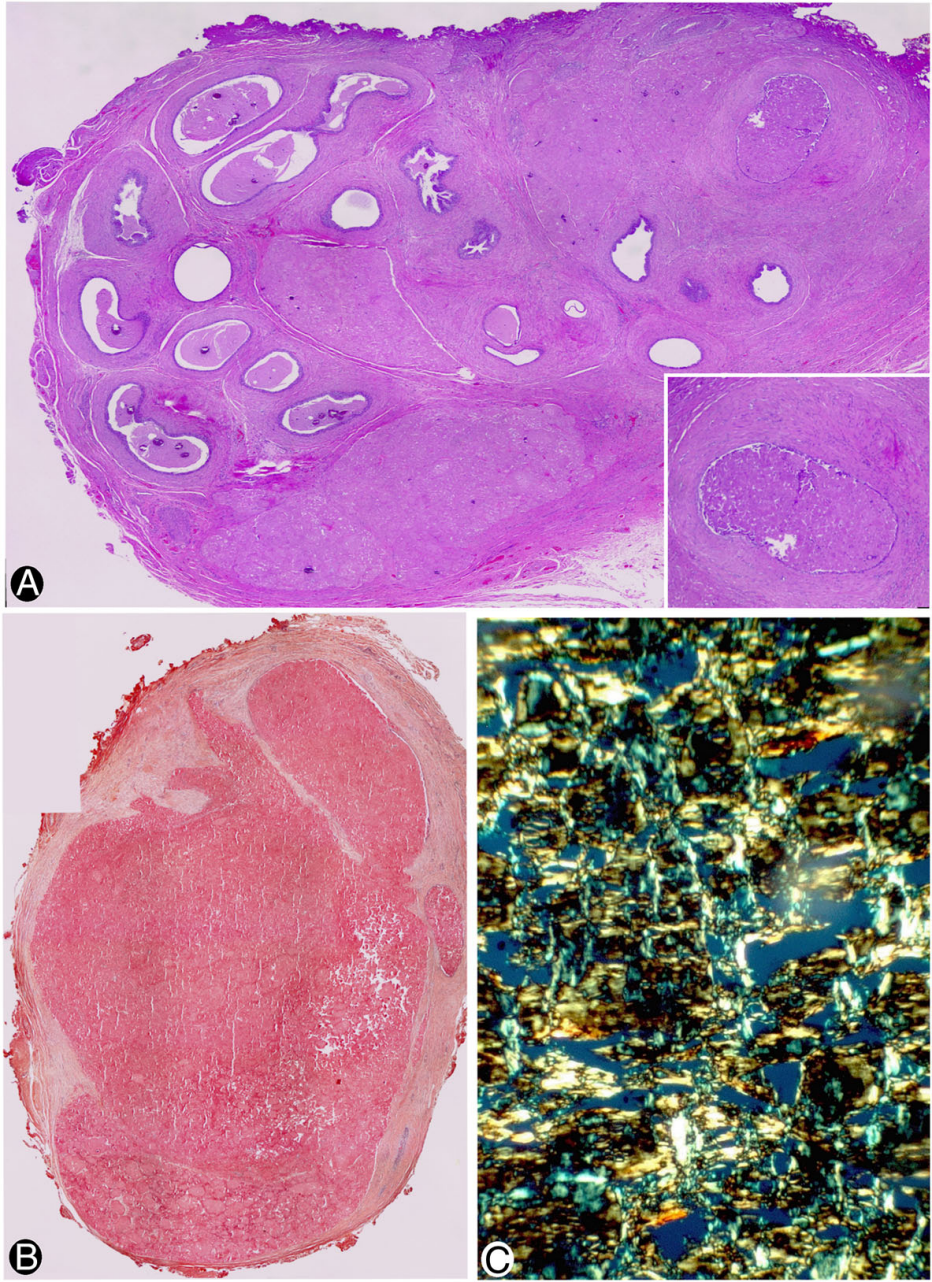
Amyloid deposits in the epididymes. **a** Eosinophilic amyloid deposits are observed in an H&E stained section. Insert: a zone of deposits in the epididymal lumen. **b** Congo red positivity. **c** Yellow-green birefringence under polarized light. **a** corresponds to case 1, and **b** and **c** to case 2. **a** and **b**: ×10 (insert in A: ×20). **c**: ×120

**Fig. 2:**
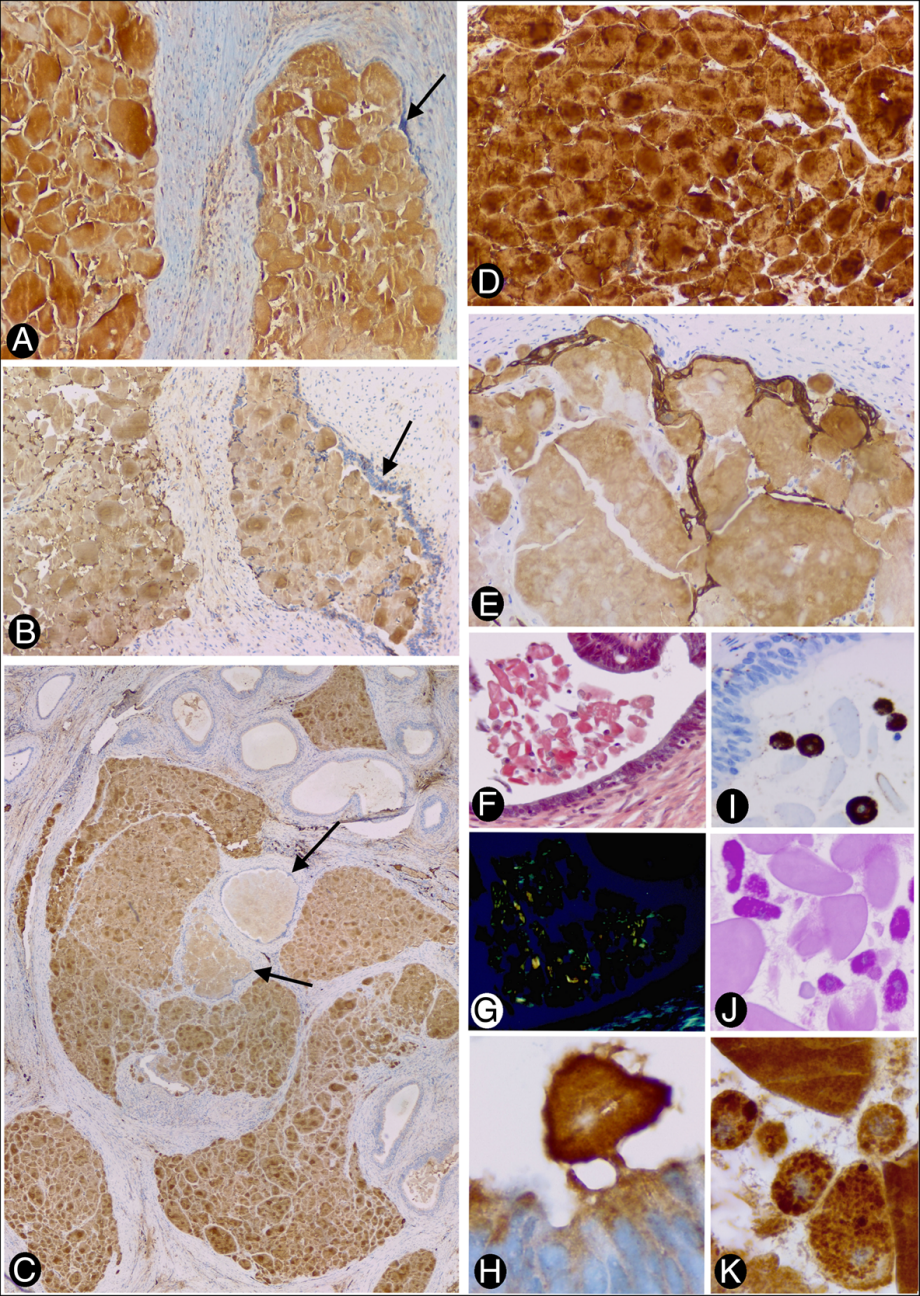
Immunohistochemical expression and distribution of amyloid deposits (**a** to **e**), and characteristics of free bodies and macrophages in other regions of the epididymal lumen (**f** to **k**). Expression in the amyloid deposits of transthyretin (**a**), light chain kappa (**b**) and lambda (**c**), amyloid P (**d**) and pan CK AE1 AE3 (**e**) is observed. Note the presence of epithelium-lined (*arrows*) (intraluminal) and non-epithelium-lined (interstitial) amyloid deposits. In C, the intraluminal and interstitial deposits are organized in a similar convoluted path to that of the epididymal tubule. In E, residual pan CK AE1 AE3+ epithelial cell bands persist in the periphery of the interstitial deposits. In other regions of the epididymal lumen, free amyloid bodies in the lumen associated with vesicles, particles and filaments are present (**f** to **h**). Note Congo red positivity (**f**) with yellow-green birefringence (**g**) and immunohistochemical expression of amyloid P (**h**). Intraluminal CD68 positive macrophages (**i**) showing PAS positive intracytoplasmic granules (**j**), which express amyloid P (**k**), are also observed. **a**, **b**, **d** and **e** correspond to case 2. **c** and **f** to **k** correspond to case 3. **a**, **b**, **d** and **e**: ×120, **c**: ×10, **f**, **g**, I and **j**: ×320, **h** and **k**: ×480

In H&E stained sections, amorphous hyaline eosinophilic deposits were observed (Fig. 1a). The deposits showed Congo red positivity (Fig. 1b), with yellow-green birefringence under polarized light (Fig. 1c), and irregular PAS positivity. Immunohistochemical expression of transthyretin (Fig. 2a), light chains kappa (Fig. 2b) and lambda (Fig. 2c), and amyloid P (Fig. 2d) was observed. Pan cytokeratin (CK) AE1 AE3 also showed irregular positivity in the amyloid deposits (Fig. 2e). There was no immunoreactivity for amyloid A, and no amyloid deposits were seen in blood vessel walls. Spermatozoa were absent.

### Distribution of amyloid deposits

Amyloid deposits were observed in the lumen of the convoluted epididymal tubule and in several lumps in the interstitium (Figs. 1a and 2a to c), showing similar immunohistochemical expression in both locations. On occasion, several separate aggregates of amyloid deposits were organized in a similar convoluted path to that of the epididymis (Fig. 2c).

The distribution and quantity of intratubular amyloid bodies varied depending on the section of the tubule. Thus, they were scarce and free in the lumen of some tubular sections of the epididymis, but numerous in others, where they were densely grouped, obliterating and distending the epididymal lumen (Figs. 1a and 2a to c). The free bodies in the lumen showed Congo red positivity (Fig. 2f), with immunofluorescence under polarized light (Fig. 2g) and amyloid P expression (Fig. 2h), and were associated with other materials, including vesicles, particles, filaments and small dense bodies. Intraluminal CD68+ macrophages (Fig. 2i) were also observed with intracytoplasmic PAS+ granules (Fig. 2j), which expressed transthyretin and amyloid P (Fig. 2k, corresponding to amyloid P).

The interstitial amyloid deposits formed aggregates, ranging from small to large interstitial masses (Figs. 1a, b and 2a to c).

### Relationship between intratubular and interstitial amyloid deposits

**Fig. 3:**
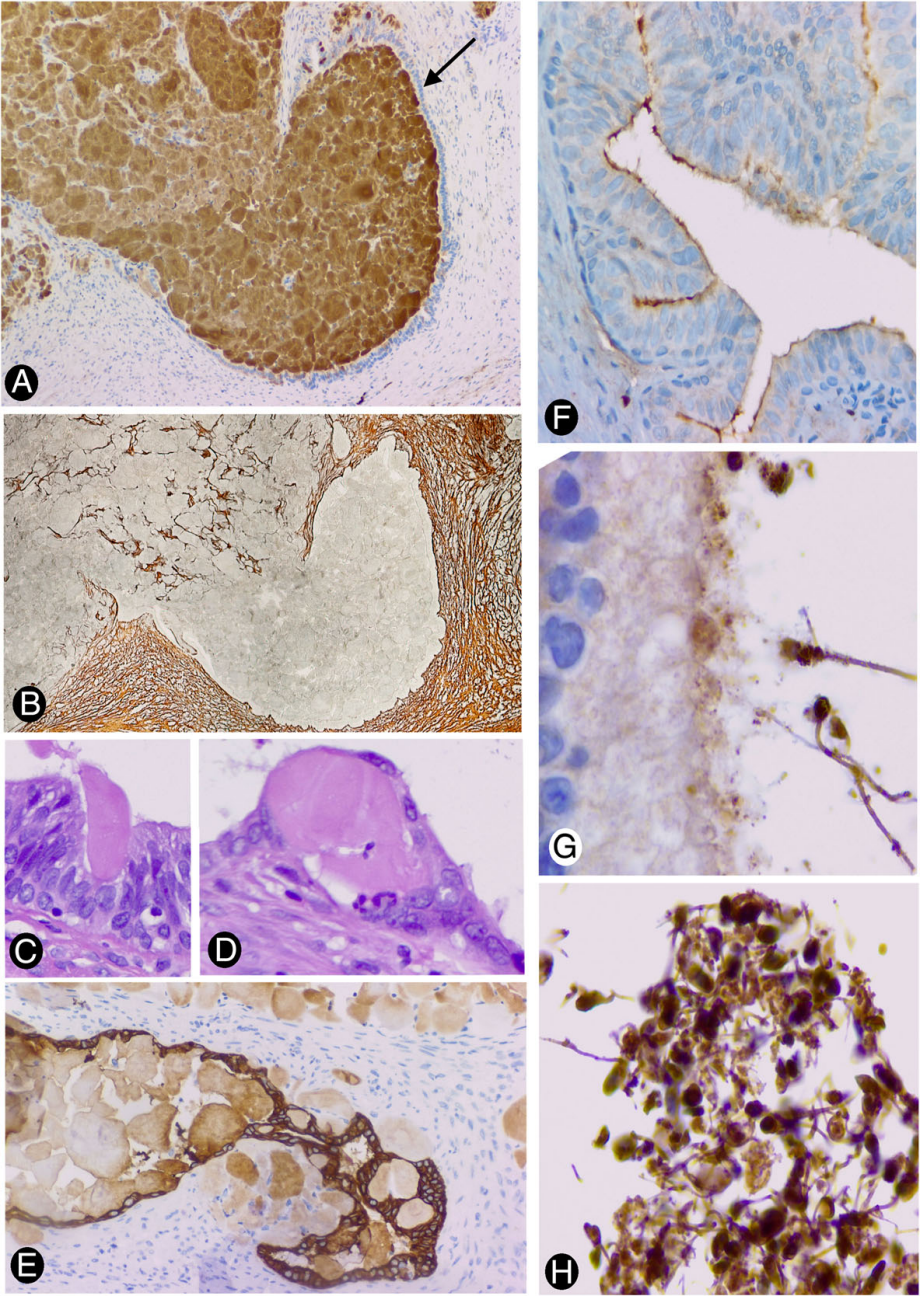
Relationship between intratubular and interstitial amyloid deposits (**a** to **d**), and detection of amyloids in normal epididymis (**e** to **g**). **a**: Epithelium-lined (*arrow*) (intraluminal) and non-epithelium-lined (interstitial) zones of an amyloid deposit are observed in continuity. **b**: A reticulin network in the interstitial zone but not in the luminal zone of the amyloid deposit is observed. **c** to **e**: Epithelial folds with degenerative phenomena are observed surrounding small portions of intraluminal amyloid deposits, which are partially incorporated in the interstitium. In normal epididymis, expression of transthyretin (**f**) and amyloid P (**g**) is observed in the apical surface of the epididymal epithelium. Strong expression of amyloid P is also shown in spermatozoa (**g** and **h**). **a**: transthyretin immunostaining. **c** and **d**: H&E staining. **e**: pan CK AE1 AE·immunostaining. **a**, **b**, **e** and **f**: ×120; **c** and **d**: ×320; **g** and **h**: ×340

Frequently, the luminal and interstitial deposits were in continuity and were therefore partially lined by epithelium (Fig. 3a), which showed pan CK AE1 AE3 and epithelial membrane antigen (EMA) expression. Residual epithelial bands were even observed on the surface of larger interstitial deposits (Fig. 2e). The intratubular and interstitial zones in these confluent deposits were not only differentiated by the presence or absence of epithelial coating, but also by the existence of other components within the deposits. A reticulin network, and CD34+ and/or αSMA+ stromal cells were observed in interstitial but not in luminal zones of the deposits (Fig. 3b, corresponding to the reticulin network). Moreover, epithelial folds with degenerative phenomena surrounded occasionally portions of intraluminal amyloid deposits, which were partially incorporated in the interstitium (Fig. 3c to e).

### Detection of amyloids (with tested expression in epididymal amyloidosis) in normal epididymides

In the epididymides surgically obtained from neighbouring pathologic processes, transthyretin (Fig. 3f) and amyloid P (Fig. 3g) were expressed in the apical surface of the epithelium. Amyloid P also showed strong expression in spermatozoa (Fig. 3g and h). Occasional macrophages with PAS and amyloid P positive bodies were seen.

## Discussion

We report three cases of localized amyloidosis in the epididymis, two clinically detected as a nodular mass and the other obtained together with a neighbouring pathologic process (para-testicular liposarcoma). Although localized amyloidosis has been described in several locations of the male reproductive system, which contribute to the transport, maturation and/or required fluid medium of spermatozoa [1–9], to the best of our knowledge, this is the first description of localized epididymal amyloidosis. Awareness of the existence of epididymal amyloidosis and of its presentation as small nodules in the epididymis, as well as knowledge of its histopathology, is of interest in clinical and pathological differential diagnoses, including tumours. Moreover, the characteristics of the lesion support the initial development of amyloid deposits in the epididymal lumen, where a specific proteome occurs [10–12], and non-pathological functional amyloids and mechanisms of protein aggregation control take place [13–15]. Below, we examine these issues.

Our cases were seen in patients aged 67 and over. Although this type of amyloidosis could be regarded as a senile form, larger series are needed to confirm this possibility.

Our observations indicate an initial deposit of amyloid in the epididymal lumen, with subsequent passage to the interstitium. The findings that support this sequence are as follows: a) densely grouped deposits in some sections of the epididymis occupy both the lumen and the interstitium, after distention of the epididymal lumen and partial epithelial disruption, b) presence of epithelial folds with degenerative phenomena, surrounding portions of intraluminal amyloid deposits, which are partially incorporated in the interstitium, c) epithelial strips remain on the surface of some large interstitial amyloid masses, d) several distinct aggregates of amyloid deposits are organized in a similar convoluted path to that of the epididymis, and e) the deposits occupying both the interstitium and the lumen appear with and without reticulin networks and/or stromal cells, respectively.

Both intraluminal and interstitial deposits in the epididymis show amyloid characteristics, including positivity for Congo red with yellow-green birefringence under polarized light. In our observations, apparent negativity for amyloid A, any evidence of systemic amyloidosis, paraproteinemia or underlying plasma cell dyscrasia, and the absence of amyloid deposits involving vascular walls support an organ-limited deposition of heterogeneous amyloids, including light chains κ and λ, and transthyretin. Amyloid P was also present. However, our immunohistochemical results were obtained in paraffin-embedded tissue blocks, and amyloid deposits can contain several misfolded proteins. Therefore, a more specific characterization of the proteome and misfolded proteins requires further study (see below).

The onset of amyloid deposits in the epididymal lumen may be related to our results showing amyloid P (involved in the deposition, stabilization and persistence of amyloid) and transthyretin (a transport protein in the serum and cerebrospinal fluid) expression in the apical surface of the normal epithelial cells of the epididymis. Moreover, amyloid P showed strong immunoreactivity in the spermatozoa, which concurs with the observations of others authors [16]. Likewise, immunoreactivity for pan CK AE1 AE3 also concurs with the presence of keratins in the sperm proteome [17].

The comparison with amyloidosis described in other locations of the male reproductive system that contribute to the transport, maturation and/or required fluid medium of spermatozoa, mainly seminal vesicles, indicates that the deposits have a different location: initially intraluminal with subsequent passage to the interstitium in the epididymis, and predominantly sub-epithelial in the seminal vesicles [1–9], as occurs in choroid plexus amyloidogenic papillomas [18]. The mechanisms to explain these differences in the polarization of the deposits require further studies. Likewise, the positive reaction for light-chain antibodies has been reported for several cases of localized amyloidosis in the urogenital tract [19, 20], and the expression of light chains κ and/or λ in localized amyloidosis in other regions is not exceptionally rare [21].

The unique and distinctive location of amyloid deposits in the epididymal lumen, with subsequent passage to the interstitium, could depend on the peculiar functions of this anatomical region, in which proteomic studies show the more sequentially modified milieu of the body [10–12]. Indeed, the epididymis actively participates in the maturation, protection and acquisition of motility and fertility of spermatozoa by synthesis, secretion and post-transitional modifications of important molecules, including a high concentration of several hundred proteins, most of which are actively secreted by the epididymal epithelium (for review, see [12]). Spermatozoa are dependent on this extracellular environment, since their DNA is highly compacted, making processes of transcription and translation impossible [22–24]. Epididymosomes (vesicles present in the epididymis) are involved in the acquisition of new sperm proteins during epididymal transit [22–24]. Some of these proteins may form functional amyloids, and in vitro studies have shown amyloid formation in this unique milieu. Thus, this milieu comprises cystatin-related epididymal spermatogenic members (CRES), which pass from monomeric forms in the proximal caput region to an aggregated amyloid state in the distal caput region [15, 25], and amyloidogenic prion protein is present in epididymosomes and associated with hydrophobic proteins in lipophilic complex [26–28]. Likewise, concentration of luminal content occurs in the epididymis (more than 90% of the fluid is removed from the epididymis) [29], facilitating macromolecular crowding, and protein misfolding and aggregation. However, in the epididymis, amyloids act without causing pathology, due to the mechanisms of extracellular quality control [13, 14, 25]. This control includes: a) ubiquitin-dependent proteolysis (classically considered as an intracellular quality control system and currently as also having extracellular functionality in sperm quality control) [30], b) chaperones (involved in the prevention of protein aggregation), since chaperone clusterin is found in soluble high molecular mass lipophilic complex present in the lumen of the epididymis during sperm maturation (around 30% of total epididymal secretion [11]), and c) transglutaminase, which prevents the formation of amyloid-type aggregates of CRES in the epididymis by post-translational modifications (transglutaminase cross-linking of cystatin CRES - [25]). Therefore, the onset of amyloidosis deposits in the lumen of the epididymis, with subsequent passage to the interstitium, suggests a disturbance of the mechanisms mentioned above, mainly of extracellular (intraluminal) functional amyloid control.

While intracellular, post-translational quality control systems to repair or remove misfolded proteins have been well studied, extracellular mechanisms of folding control of secreted proteins are not well described, except in the lumen of the epididymis [13, 14]. These mechanisms in the epididymis have been considered highly significant for understanding the misfolded protein formation involved in some pathological processes, including Alzheimer’s disease, cerebral angiopathies, and type II diabetes [13, 14]. In this way, amyloid deposits in the epididymis may provide a substrate to explore not only the alteration of the reproductive function, but also the mechanism of extracellular protein misfolding control in several diseases. Thus, new studies in epididymal amyloidosis are required to explore the functional amyloids outlined above and the molecules that act in their extracellular quality control.

## Conclusion

We describe the presence of localized amyloidosis in the epididymis for the first time. The initial intraluminal formation is particularly interesting because it may also be the pathological expression of amyloid in an anatomical region with critical functions during sperm maturation, including the uniqueness of the human epididymal proteome and several molecular pathways, such as those involved in specific intraluminal functional amyloids and their quality control.
